# Development and validation of a nomogram to predict mortality of patients with DIC in ICU

**DOI:** 10.3389/fmed.2024.1425799

**Published:** 2024-07-09

**Authors:** Qingbo Zeng, Qingwei Lin, Lincui Zhong, Longping He, Nianqing Zhang, Jingchun Song

**Affiliations:** ^1^Intensive Care Unit, The 908th Hospital of Chinese PLA Logistic Support Force, Nanchang, China; ^2^Intensive Care Unit, Nanchang Hongdu Hospital of Traditional Chinese Medicine, Nanchang, China

**Keywords:** nomogram, Lasso-Cox regression, disseminated intravascular coagulation, prediction, short-term mortality, intensive care unit

## Abstract

**Background:**

Disseminated intravascular coagulation (DIC) is a devastating condition, which always cause poor outcome of critically ill patients in intensive care unit. Studies concerning short-term mortality prediction in DIC patients is scarce. This study aimed to identify risk factors contributing to DIC mortality and construct a predictive nomogram.

**Methods:**

A total of 676 overt DIC patients were included. A Cox proportional hazards regression model was developed based on covariates identified using least absolute shrinkage and selection operator (LASSO) regression. The prediction performance was independently evaluated in the MIMIC-III and MIMIC-IV Clinical Database, as well as the 908th Hospital Database (908thH). Model performance was independently assessed using MIMIC-III, MIMIC-IV, and the 908th Hospital Clinical Database.

**Results:**

The Cox model incorporated variables identified by Lasso regression including heart failure, sepsis, height, SBP, lactate levels, HCT, PLT, INR, AST, and norepinephrine use. The model effectively stratified patients into different mortality risk groups, with a C-index of >0.65 across the MIMIC-III, MIMIC-IV, and 908th Hospital databases. The calibration curves of the model at 7 and 28 days demonstrated that the prediction performance was good. And then, a nomogram was developed to facilitate result visualization. Decision curve analysis indicated superior net benefits of the nomogram.

**Conclusion:**

This study provides a predictive nomogram for short-term overt DIC mortality risk based on a Lasso-Cox regression model, offering individualized and reliable mortality risk predictions.

## Introduction

Disseminated intravascular coagulation (DIC) frequently occurs in critically ill patients admitted to the intensive care unit (ICU) and is associated with high mortality. DIC is a severe syndrome characterized by systemic activation of coagulation, and plenty of thrombin with fibrin deposition in the micro- and macrovascular systems (1, 2), which is provoked by various underlying diseases, such as serious infections, trauma, malignancies, and liver disease. The complex pathophysiology of DIC involves impairment of anticoagulant mechanisms, uncontrolled activation of the tissue factor pathway, and suppression of fibrinolysis (3, 4). These changes can lead to life-threatening thrombosis and bleeding event, as well as organ dysfunction, which will substantially increase mortality (5, 6). Therefore, the key to therapy for DIC is to control activation of blood coagulation and decrease bleeding and thrombosis risk. Currently, the clinically therapeutic strategies for DIC are limited and prognosis is often poor although treatment of the underlying condition may improve DIC (7). Obviously, it is necessary to identify its potential death risk factors if DIC occurred.

Close monitoring of coagulation parameters is required for patients with DIC given their severely abnormal and dynamic laboratory values and clinical status. However, quantification of mortality risk based on the large volume of data available in electronic health records poses a challenge for ICU physicians. Rapid identification of high-risk patients and timely intervention remain clinically difficult.

Recent advancements in prognostic and diagnostic tools, such as nomogram and artificial intelligence algorithms, have demonstrated efficacy in a range of medical disciplines (8, 9). Previous studies have shown that the models established by these methods can be used in the survival analysis of multiple diseases and show more promising performance than traditional illness severity scoring systems (10–14). Regrettably, prognostic models specifically catering to overt DIC patients within ICU settings remain conspicuously absent. Therefore, this study aims to construct a new prediction model based on the Lasso-Cox method to predict the survival probability of critically ill patients with overt DIC during hospitalization accurately, which would be helpful to provide timely management of overt DIC.

## Methods

### Source of data

This retrospective observational cohort study utilized data from the MIMIC-III version 1.4 and MIMIC-IV version 2.0 databases. Both MIMIC-III and MIMIC-IV are openly available critical care databases containing ICU patient data from Boston’s Beth Israel Deaconess Medical Center. Permission to extract data from these databases was granted following a rigorous deidentification process overseen by the Harvard Medical School’s Ethics Review Board and the Massachusetts Institute of Technology (Record ID: 11763035). We also included the patients from the 908th Hospital of Chinese PLA Logistic Support Force as the external validation set. The institutional review board of the 908th Hospital of Chinese PLA Logistic Support Force approved our study and waived the requirement for informed consent because of the retrospective nature of the present study.

### Study population and data extraction

The following data were obtained from the MIMIC-III, MIMIC-IV, and the 908th Hospital databases: (1) demographic data; (2) comorbidities, including Atrial fibrillation, sepsis, congestive heart failure, hypertension, chronic obstructive pulmonary disease, and diabetes; (3) outcomes, including ICU stay time, 7-day mortality, and 28-day mortality; (4) severity score, including simplified Acute Physiology Score II (SAPSII), sequential organ failure assessment (SOFA) score; (5) mean value of vital signs and the laboratory test value within 24 h of admission to the ICU; and (6) treatment measures (renal replacement therapy). Data were extracted using PostgreSQL program (version 12). Adult DIC patients (≥18 years) were included as defined according to the ISTH criterion (15): platelet count<100*10^9^/L = 1 point, <50*10^9^/L = 2 points; PT prolongation of >3 s = 1 point, >6 s = 2 points; FIB<1.0 g/L = 1 point; a moderate increase in FDP or D-dimer levels = 2 points, a strong increase = 3 points. Overt DIC was diagnosed if a total score was not less than 5. Exclusion criteria were: (1) age < 18 years; (2) pregnant women; (3) patients with congenital coagulopathy; (4) the coagulation function was frequently affected by the pathologic states of tumors and the chemotherapy agent used; and (5) dying or leaving within 24 h after ICU admission.

### Statistical analysis

Categorical variables were compared using the chi-squared test, while continuous variables were analyzed using the Student’s *t*-test or the Wilcoxon–Mann–Whitney test. Lasso regression was employed to identify significant risk factors. The prediction model including variables screened by Lasso regression were constructed based on Cox regression. The performance of predictive nomogram was evaluated by the C-index and calibration curve. DCA analysis was applied to assess the clinical usefulness. Clinical applicability was assessed using the Kaplan–Meier method. All of statistical analyses were conducted with R software v4.1.1 and SPSS 27.0 software. Variables missing more than 5% of values were handled using the random forest (RF) method, based on the“randomForest”package of R. For variables missing less than 5% of values, median imputation was used. Variables with more than 30% missing values were excluded. A *p* value of <0.05 was considered statistically significant.

## Results

### Characteristics of the study participants

**Figure 1:**
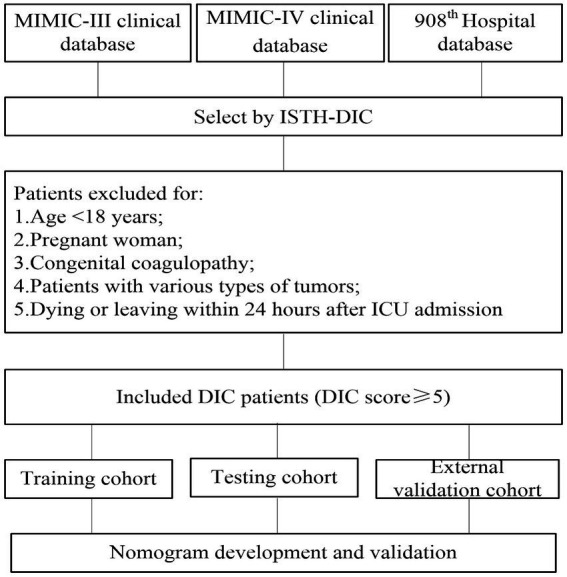
Flowchart of patient inclusion in the study.

The study integrated 39 routinely available variables from ICU patients, comprising of 32 continuous and seven categorical variables (see Table 1). The correlative data of patients in the MIMIC-III were extracted. After data preprocessing and removal of samples with missing values, the MIMIC-III database contributed 148 patients. Employing the same inclusion and exclusion criteria, 386 patients were drawn from MIMIC-IV and 142 additional patients were randomly selected from the 908th Hospital (Figure 1).

**Table 1:** Comparison of clinical data.

Variables	Training set (*N* = 148)	Testing set (*N* = 386)	External testing set (*N* = 142)
Male, *n* (%)	83 (56.1)	213 (55.2)	86 (60.6)
Age, (years)	63 (51–75)	58 (50–72)	60 (44–75)
Weight (kg)	78 (65–96)	79 (67–98)	75 (59–95)
Height (cm)	169 (163–178)	170 (160–178)	168 (161–176)
Hypertension, *n* (%)	50 (33.8)	82 (21.2)	47 (33.1)
Diabetes, *n* (%)	46 (31.1)	121 (31.3)	45 (31.7)
COPD, *n* (%)	2 (1.3)	7 (1.8)	3 (2.1)
Atrial fibrillation, *n* (%)	42 (28.4)	134 (34.7)	39 (27.5)
Heart failure, *n* (%)	44 (29.7)	90 (23.3)	36 (25.3)
Sepsis, *n* (%)	78 (52.7)	159 (41.2)	57 (40.1)
Trauma, *n* (%)	3 (2.0)	21 (5.4)	5 (3.5)
Temperature (°C)	36.7 (36.2–37.5)	36.7 (36.4–37.2)	36.6 (36.1–37.3)
RR (rate/min)	21 (18–25)	20 (17–23)	20 (18–25)
HR (rate/min)	93 (80–107)	88 (79–104)	91 (76–105)
SBP (mmHg)	110 (102–122)	107 (102–117)	116 (96–135)
DBP (mmHg)	60 (53–67)	58 (52–65)	65 (56–77)
MBP (mmHg)	74 (67–83)	73 (67–79)	77 (65–86)
WBC (×10^9^/L)	10.3 (6.3–14.5)	10.4 (6.4–15.8)	12.1 (8.1–16.5)
RBC (×10^12^/L)	3.1 (2.8–3.6)	2.8 (2.5–3.4)	3.0 (2.4–3.5)
HB (g/L)	94 (83–109)	90 (77–104)	90 (72–111)
HCT (%)	28 (25–31)	28 (24–32)	28 (22–33)
PLT (×10^9^/L)	92 (49–199)	93 (58–173)	68 (38–107)
ALT (U/L)	34.5 (20.3–103.8)	34.0 (20.0–74.8)	84.9 (40.6–407.7)
AST (U/L)	55.5 (30.0–159.8)	59.0 (38.0–137.3)	60.1 (28.3–323.0)
Tbil (mg/dL)	1.5 (0.6–4.5)	1.9 (0.9–4.9)	0.8 (0.5–1.6)
BUN (mg/dL)	32 (19–51)	29 (18–46)	33 (21–53)
Cr (mg/dL)	1.4 (0.9–2.4)	1.2 (0.9–2.0)	1.2 (0.8–1.7)
Glu (mg/dL)	117 (101–150)	120 (99–163)	119 (103–161)
PT (s)	16.8 (14.8–19.7)	20.7 (18.7–26.7)	21.9 (18.7–27.5)
INR	1.5 (1.3–2.0)	1.9 (1.7–2.5)	1.8 (1.5–2.1)
FIB (g/L)	3.36 (1.85–5.60)	1.99 (1.39–3.43)	1.36 (0.81–2.57)
D-dimer (mg/L)	6.32 (4.04–9.61)	2.46 (1.12–6.74)	10.66 (4.98–20.49)
PH	7.33 (7.26–7.42)	7.38 (7.28–7.44)	7.34 (7.26–7.42)
PaO2 (mm)	102 (78–130)	137 (76–291)	125 (71–174)
PaCO2 (mm)	39 (33–44)	38 (32–44)	36 (29–42)
Lactate (mmol/L)	2.0 (1.4–3.0)	2.4 (1.6–4.0)	4.3 (2.4–7.4)
CRRT, *n* (%)	39 (26.4)	78 (20.2)	23 (16.2)
Epinephrine, *n* (%)	4 (2.7)	9 (2.3)	6 (4.2)
Norepinephrine, *n* (%)	62 (41.9)	194 (50.2)	79 (55.6)
SAPSS II	50 (36–61)	45 (36–56)	47 (35–58)

A total of 676 overt DIC patients were included. Of these, 32.5% experienced short-time mortality. The patient baseline characteristics are detailed in Table 1. The median patient age was 60, with 382 (56.5%) being male. 179 (26.5%) individuals were diagnosed with hypertension, 215 (31.8%) with atrial fibrillation, 170 (25.1%) with heart failure, 294 (43.5%) with sepsis, 212 (31.4%) with diabetes, and 29 (4.3%) with trauma. There were 140 (20.7%) patients who received continuous renal replacement therapy (CRRT).

### Variables selection

**Figure 2:**
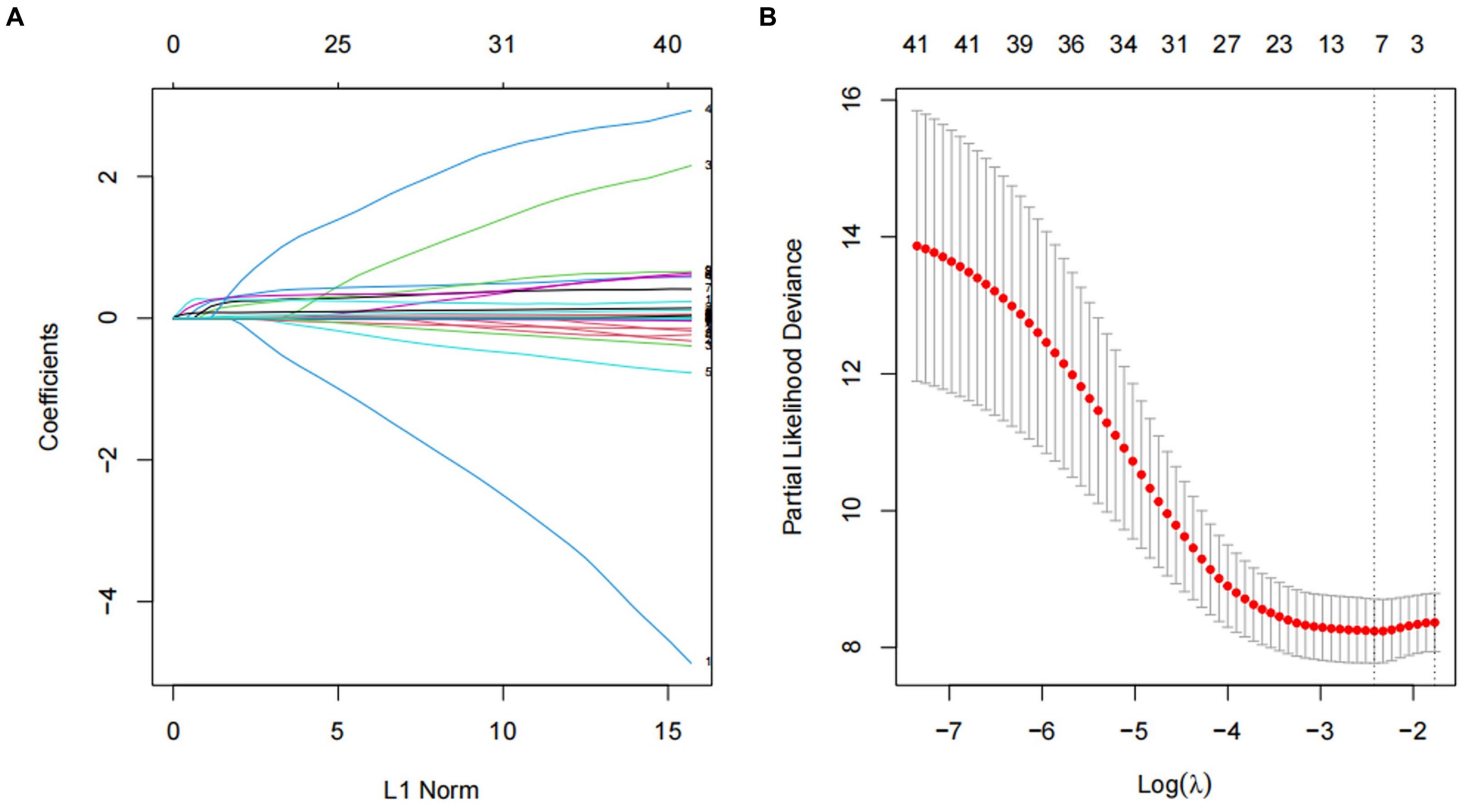
Screening of variables based on Lasso regression. **(A)** The variation characteristics of the coefficient of variables; **(B)** the selection process of the optimum value of the parameter λ in the Lasso regression model by cross-validation method.

We utilized Lasso regression to identify significant predictors and the variation characteristics of these variables were shown in Figure 2. This analysis indicated heart failure, sepsis, height, SBP, lactate, HCT, PLT, INR, AST, and norepinephrine as relevant variables. Finally, Cox regression model was further established based on variables screened by Lasso regression (Table 2).

**Table 2:** Cox regression to predict prognosis based on Lasso regression.

Variables	HR (95%)	Coefficient	*p value*
Heart failure	1.74 (0.97–3.13)	0.550	0.062
Sepsis	1.86 (0.99–3.49)	0.727	0.053
Height	0.99 (0.97–1.00)	−0.015	0.116
SBP	0.99 (0.97–1.02)	−0.005	0.719
Lactate	1.09 (0.98–1.21)	0.101	0.119
HCT	1.07 (1.01–1.14)	0.063	0.019
PLT	0.99 (0.98–1.02)	−0.003	0.031
INR	1.60 (1.17–2.19)	0.499	0.003
AST	1.02 (1.00–1.03)	0.002	0.042
Norepinephrine	1.53 (0.82–2.85)	0.424	0.183

### Development of a multivariate prognostic nomogram

**Figure 3:**
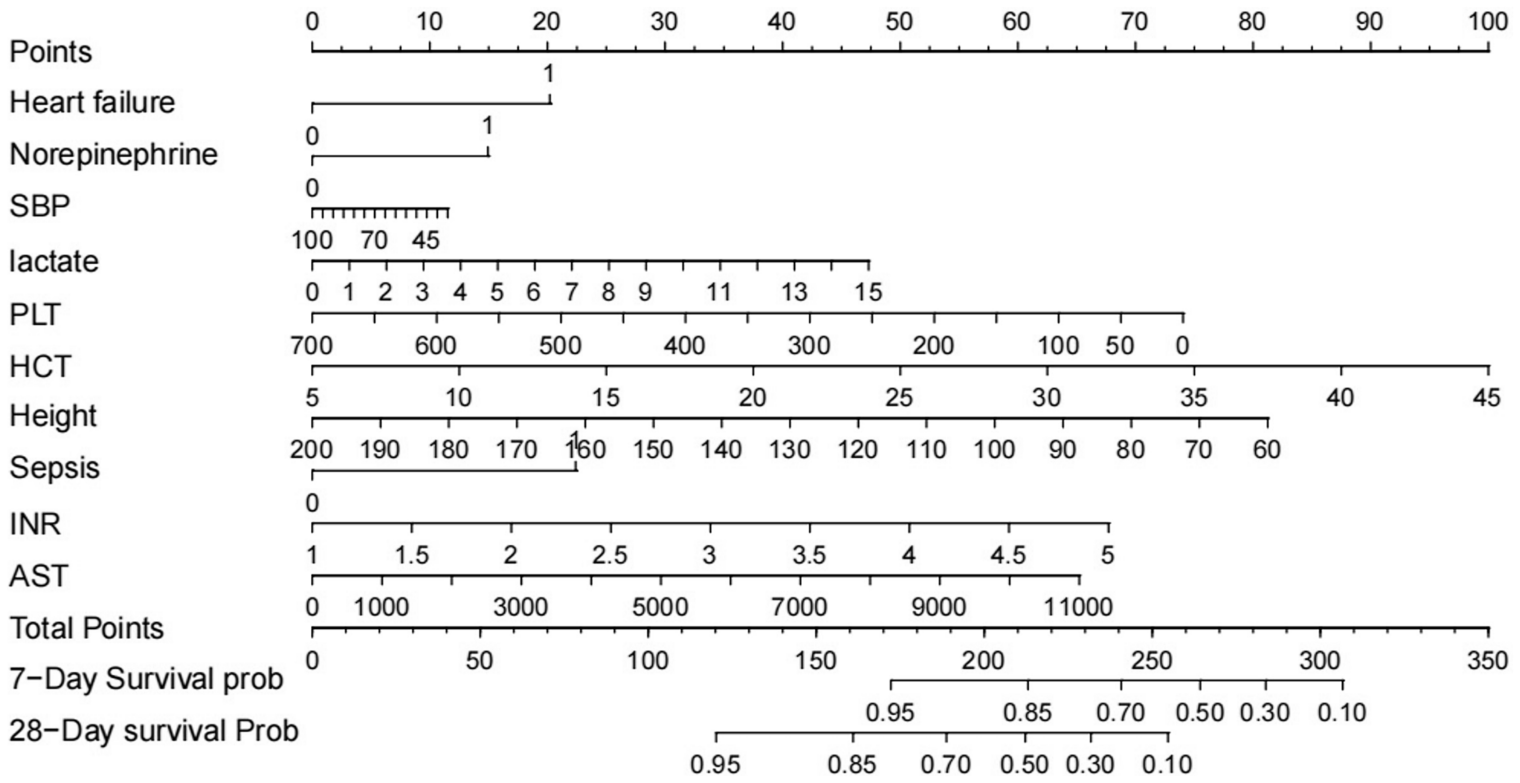
Nomogram to predict the risk of short-term mortality of patients with DIC.

The patients were divided into the training, testing, and external validation sets. The training set was used to develop the predictive model, while the testing and external validation sets were applied to validate. On the basis of the LASSO regression, a prognostic nomogram was constructed, integrating the nine significant factors (Figure 3). This nomogram was subsequently validated using both testing and external validation sets.

### Validation of the prediction nomogram

**Figure 4:**
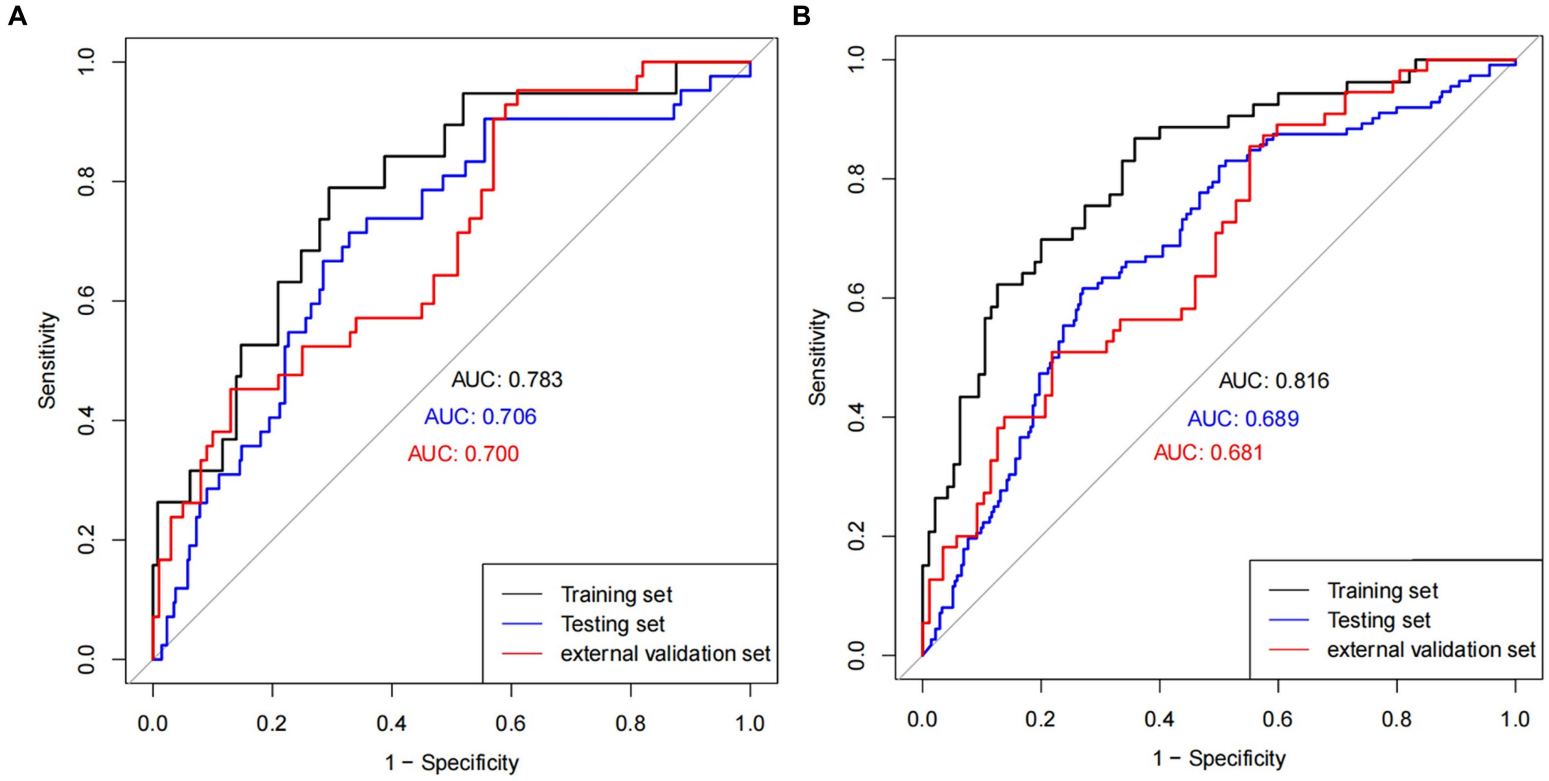
ROC plot. ROC curves in **(A)** 7 days and **(B)** 28 days in training, testing, and external validation cohort population.

The nomogram demonstrated good accuracy for predicting overt DIC patient short-term mortality. The nomogram exhibited the C-index of 0.81 and 0.77 for predicting 7- and 28-day mortality in the training set, and the C-index of 0.63 and 0.67 in the testing set. In the external validation set, C-index was 0.65 and 0.68 in predicting 7- and 28-day mortality. The AUCs for the 7-day mortality probabilities were 0.78, 0.71, and 0.70 in the training, testing, and external validation sets, while the AUCs for the 28-day mortality probabilities 0.82, 0.69, and 0.68, respectively (Figures 4A,B).

### Calibration and clinical application of nomogram

**Figure 5:**
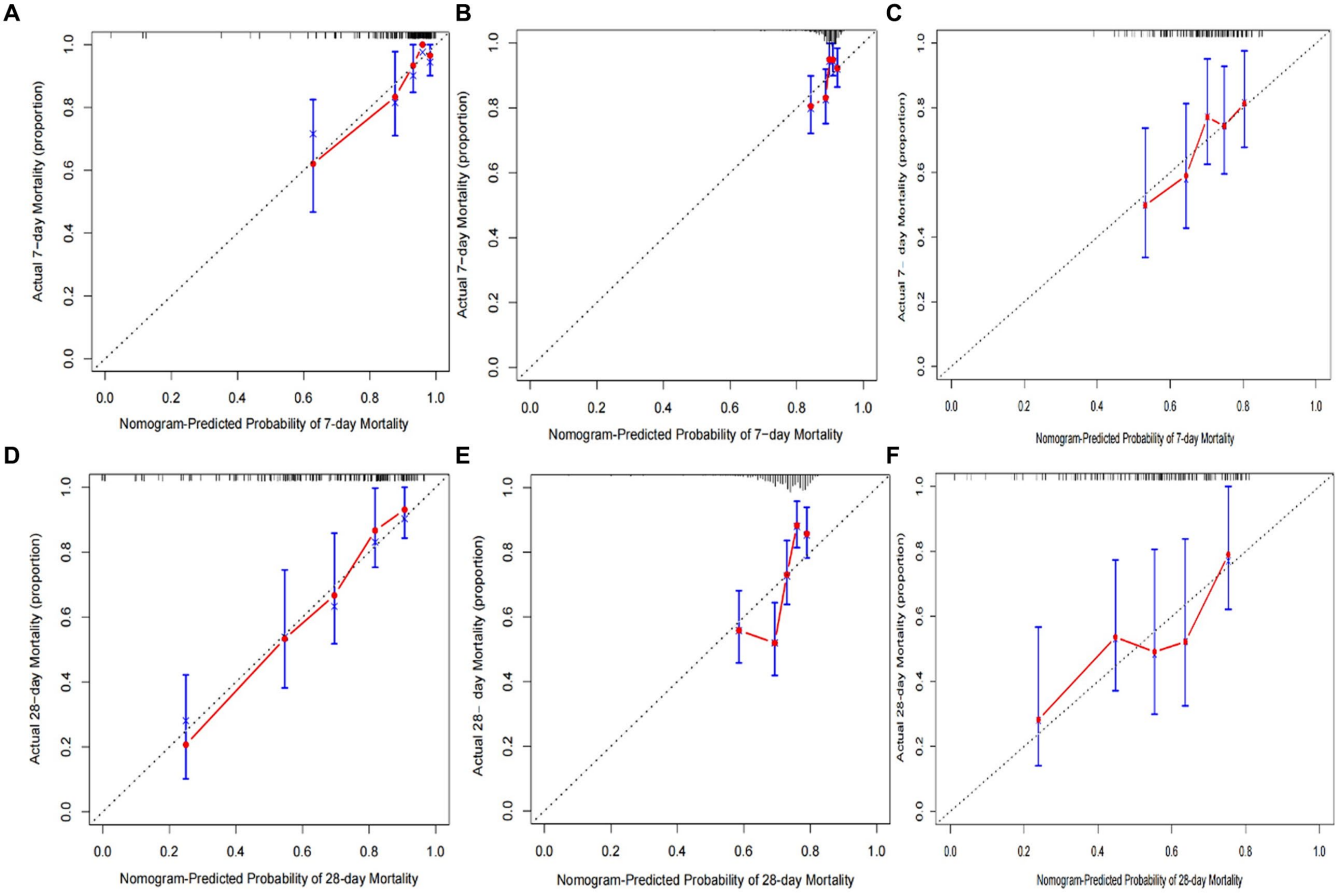
Calibration plots of predicted 7- and 28-day mortality based on Cox regression modeling in the training, testing, and external validation sets **(A–F)**.

**Figure 6:**
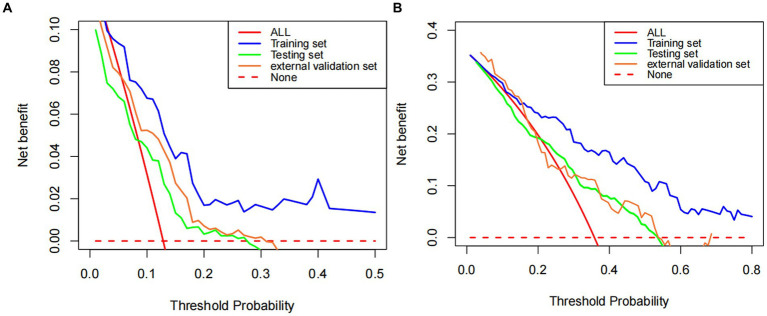
DCA of predicted 7- and 28-day mortality based on Cox regression modeling **(A,B)**.

The calibration curves of the nomogram established for predicting mortality of 7 and 28 days showed a good consistency between observed and predicted outcomes in the training, testing, and external validation sets (Figures 5A–F). In addition, the decision curve analysis displayed the prediction model provides useful prognostic information to assist clinical decision making (Figures 6A,B).

**Figure 7:**
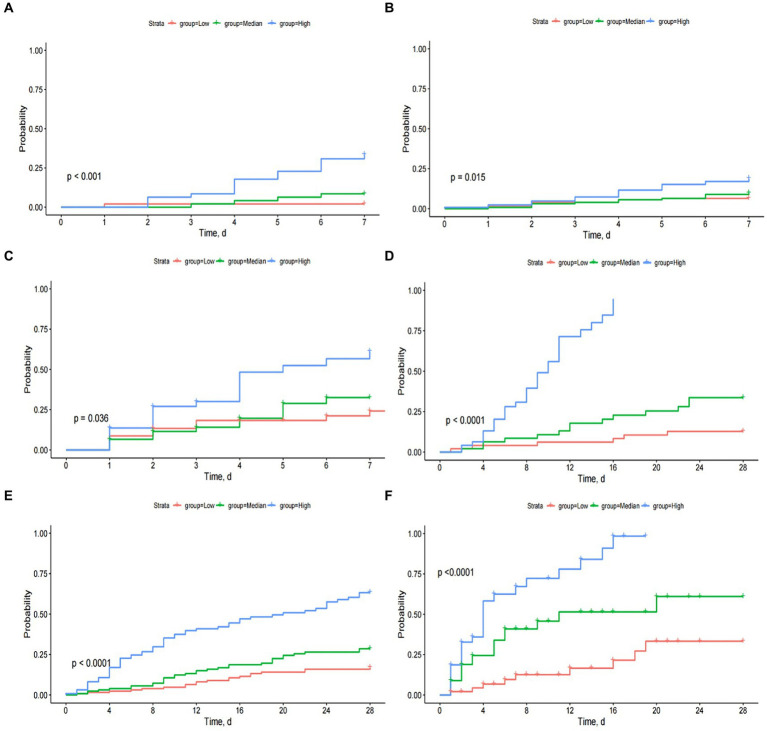
The Kaplan–Meier curves by tertiles of predicted 7- and 28-day mortality in training, testing, and external validation sets **(A–F)**.

Risk strata were generated based on tertiles of predicted 7- and 28-day mortality. K-M curves of mortality were plotted based on the risk strata in the training, testing, and external validation sets. The results demonstrated that the model can effectively stratify patients and predict 7- and 28-day mortality (Figures 7A–F).

## Discussion

Disseminated intravascular coagulation is a devastating condition and associated with high mortality. For instance, 30-day mortality rates in septic DIC have been observed to reach approximately 20%, while the 30-day mortality is up to 45% in a heterogeneous DIC population (16, 17). Therefore, we deemed 7- and 28-day mortality as suitable outcomes for this study. The assessment of the short-term death probability of overt DIC patients is an important reference for physicians to select appropriate intervention times and decide individual treatment strategies. Hence, it is necessary to analyze the risk factors affecting the outcome of overt DIC patients and establish a prognostic model to make the individualized prediction of mortality risk.

This study utilized Lasso regression to screen potential factors and establish a predictive nomogram. Lasso regression provides an advantage over univariate analysis in addressing the problem of multicollinearity among variables (18). And it was confirmed in this study that the Cox regression had a good C-index, which indicated the prediction effect of this nomogram model was better. This model integrated 10 clinically relevant variables encompassing underlying diseases (sepsis and heart failure), vasopressor use, coagulation function status (PLT and INR), hematologic condition and metabolism (HCT and Lactate), liver function status (AST), and demographic data (height and SBP). These indicators could comprehensively assess the specific situation of individuals so as to better predict the mortality risk of overt DIC. All indicators included were scored on the basis of their contribution to the prognosis, and the scores of these variables were finally summed. A vertical line at the position of total scores would be drawn to make it cross with the other two lines representing the predictive risk of death. Additionally, this model does not only incorporate patient death or survival outcomes but also the patient length of stay and survival, thus reflecting the risk of events at every stage following ICU admission. In other words, this prediction model could evaluate patients’ survival probability in the dimension of survival time.

In terms of model evaluation, we employed DCA, calibration and C-index to estimate the predictive efficacy of our model in the training, testing and external validation cohorts. These methods are widely used to assess a model’s performance (19, 20). Despite a decrease in evaluation accuracy in the testing and external validation sets, the developed Lasso-Cox regression-based prediction model remained effective in predicting 7- and 28-day prognoses. The decrease could be attributed to data heterogeneity across the three datasets. From our perspective, the ICU data from different medical centers were biased, as they were collected retrospectively and there are no restrictions on the conditions of laboratory examination equipment and the measurement methods of vital signs.

Our established predictive model facilitated clear stratification of patients into three groups based on individualized death risk. As shown in Figures 6A–F, the model could perform well in predicting short-term mortality and identify patients at high mortality risk, which indicated this nomogram had considerable predictive strength. To validate our result again, survival curves were plotted to explore whether patients with varying predicted mortality risks experienced different outcomes in the testing and external validation sets. As we expected, a higher risk of death did correspond to a poorer prognosis, reaffirming the robustness of our model. Therefore, a prediction model based on the Lasso-Cox regression model possessed a significant reference value for clinicians to identify the individual risk of mortality intuitively. In other words, the higher the mortality probability reflected in the nomogram indicated the shorter the survival time of patients, and these patients need to be treated sooner.

The present study acknowledges certain limitations. Firstly, it is a retrospective study using ICU databases, which may encompass missing data and data collection errors. Secondly, the DIC in this study is primarily attributed to sepsis, which may impact the predictive performance of our prediction model for bleeding-induced DIC. Thirdly, regarding treatment, we only examined CRRT and vasopressor use. The prognosis of overt DIC patients could also be influenced by other therapeutic measures such as plasma transfusion, cryoprecipitate transfusion, and platelet transfusion, but such data were unavailable in the MIMIC database. Finally, to validate our findings, multicenter registry and prospective studies may be necessary.

## Conclusion

The present study developed a nomogram for predicting mortality in patients with overt DIC based on the Lasso-Cox regression model. This prediction model may help ICU doctors detect overt DIC patients with a higher mortality risk ahead of time, enabling timely care and allocating appropriately medical resources to increase the overall patient population survival.

## Data availability statement

Publicly available datasets were analyzed in this study. The datasets generated and/or analyzed during the current study are available in the MIMIC-III and MIMIC-IV database, https://mimic.physionet.org/iii/ and https://mimic.physionet.org/iv/. The raw data from the 908th hospital supporting the conclusions of this article are available upon reasonable request so long as relevant patient confidentiality regulations can be followed.

## Ethics statement

The studies involving humans were approved by the institutional review board of the 908th Hospital of Chinese PLA Logistic Support Force. The studies were conducted in accordance with the local legislation and institutional requirements. Written informed consent for participation was not required from the participants or the participants’ legal guardians/next of kin in accordance with the national legislation and institutional requirements.

## Author contributions

QZ: Conceptualization, Writing – original draft, Writing – review & editing. QL: Data curation, Writing – review & editing. LZ: Data curation, Writing – review & editing. LH: Formal analysis, Writing – review & editing. NZ: Methodology, Writing – review & editing. JS: Conceptualization, Funding acquisition, Supervision, Writing – original draft, Writing – review & editing.
